# Supplementary material for: Protease‐resistant streptavidin for interaction proteomics
Source: Mol Syst Biol. 2020 May 13;16(5):e9370. doi: 10.15252/msb.20199370 (PMC7218406; doi:10.15252/msb.20199370)
Supplement: Supplementary file 1 — Expanded View Figures PDF [file MSB-16-e9370-s001.pdf]

Expanded View Figures

A

>sp|P22629|SAV_STRAV Streptavidin OS=*Streptomyces avidinii* OX=1895 PE=1 SV=1

MRKIVVAAIAVSLTTVSITASASADPSKDSKAQVSAAEAGITGTWYNQLGSTFIVTAGAD
GALTGTYESAVGNAESRYVLTGRYDSAPATDGSGTALGWTVAWKNNYRNAHSATTWSGQY
VGGAEARINTQWLLTSGTTEANAWKSTLVGHDTFTKVKPSAASIDAAKKAGVNNGNPLDA
VQQ

B

Peptide	m/z
STLVGHDTFTK	m/z = 603.3
NAHSATTWSGQYVGGAEAR	m/z = 655.3
INTQWLLTSGTTEANAWK	m/z = 1018.0

C

			Intensity: Streptavidin	Intensity: All proteins	Streptavidin (%)
BioID Low amount	Ctr_wtS	R1	4.0E+09	6.3E+09	64.09
		R2	4.0E+08	6.2E+08	63.67
		R3	4.85+09	7.9E+09	61.49
	Ctr_prS	R1	6.5E+08	4.5E+10	1.46
		R2	7.5E+08	4.5E+10	1.67
		R3	1.0E+09	5.9E+10	1.73
	BioID_wtS	R1	3.4E+09	5.4E+09	63.85
		R2	3.3E+09	5.0E+09	67.00
		R3	4.4E+09	6.8E+09	64.72
	BioID_prS	R1	6.0E+08	5.7E+10	1.06
		R2	7.1E+08	5.6E+10	1.27
		R3	6.7E+08	6.1E+10	1.11

			Intensity: Streptavidin	Intensity: All proteins	Streptavidin (%)
BioID High amount	Ctr_wtS	R1	1.2E+11	2.1E+11	60.34
		R2	1.3E+11	2.0E+11	62.34
		R3	7.3E+10	1.5E+11	49.36
	Ctr_prS	R1	1.0E+10	4.6E+11	2.25
		R2	7.7E+09	4.1E+11	1.87
		R3	9.2E+09	5.0E+11	1.85
	BioID_wtS	R1	1.5E+11	1.9E+11	77.39
		R2	1.4E+11	1.9E+11	72.01
		R3	1.2E+11	1.8E+11	66.96
	BioID_prS	R1	8.2E+09	5.1E+11	1.60
		R2	7.4E+09	5.0E+11	1.48
		R3	6.1E+09	4.6E+11	1.33

			Intensity: Streptavidin R1	Intensity: Streptavidin R2	Intensity: All proteins R1	Intensity: All proteins R2	Streptavidin (%) R1	Streptavidin (%) R2
ChIP-SICAP	wtS_HpH fractions	fr1	3.2E+07	2.5E+07	2.6E+08	2.9E+08	12.51	8.78
		fr2	5.8E+06	1.6E+07	9.0E+07	1.1E+08	6.44	14.14
		fr3	8.9E+05	8.5E+05	2.0E+08	2.3E+08	0.45	0.36
		fr4	2.1E+08	2.3E+08	5.0E+08	5.7E+08	42.87	40.53
		fr5	8.2E+07	8.9E+08	1.2E+08	1.2E+09	68.70	76.30
		fr6	1.5E+08	2.6E+08	7.5E+08	1.1E+09	19.97	24.40
		fr7	5.6E+07	6.0E+07	4.5E+08	4.6E+08	12.35	12.93
		fr8	3.9E+07	6.3E+07	1.6E+08	2.0E+08	24.56	30.83
		fr9	1.8E+07	2.4E+07	2.2E+08	3.8E+08	8.11	6.24
		fr10	1.2E+07	2.4E+07	4.5E+08	6.0E+08	2.60	4.01
	Single injection	wtS	4.1E+09	3.3E+09	8.2E+09	6.3E+09	50.24	51.84
		prS	2.6E+06	0	1.7E+10	2.2E+10	0.01	0.00

		Intensity: Streptavidin	Intensity: All proteins	Streptavidin (%)
Lipids	R1	1.9E+11	1.7E+12	9.76
	R2	1.4E+11	1.1E+12	11.44
	R3	1.4E+11	1.7E+12	7.19

		Intensity: Streptavidin	Intensity: All proteins	Streptavidin (%)
Membrane proteins	wtS_10	1.1E+11	1.6E+11	70.58
	prS_10	9.4E+06	1.4E+10	0.07
	prS_90	1.6E+08	9.6E+10	0.18

Figure EV1. Tryptic digestion of streptavidin.

A Amino acid sequence of streptavidin protein. Residues cleaved by trypsin are shown in red.
B Sequence and *m/z* of the top-3 streptavidin peptides, used to determine the extracted ion chromatogram (XIC) shown in main Fig 1E and F.
C Summary of the total iBAQ (All proteins) and streptavidin iBAQ values in all the experiments shown in main Figs 2 and 3.

© 2020 The Authors

Figure EV2. Suz12 chromatin interactors identified via prS bead ChIP-SICAP experiment and single injection or wtS bead enrichment followed by HpH fractionation.

A Experimental procedure.

B Intensity of all PRC2 complex components and streptavidin in ChIP-SICAP with wtS beads and subjected to HpH fractionation or prS bead and single injection. The error bars show standard deviation of two replicates

C Digesting mock beads prepared according to Barshop *et al* (blue) and prS (red) protocols. Intensity of streptavidin peptides (left) and XICs of the top-two streptavidin peptides (right).

D Comparing beads prepared according to Barshop *et al* with prS beads using a Suz12 ChIP-SICAP assay. Intensity of streptavidin (left). Number of identified proteins (right).

E Comparative Suz12 ChIP-SICAP between 2i and serum. Full MS spectra between 612–618 *m/z* at retention time: 64–65 min are shown. Specific precursors (*m/z*: 617.2866 and 612.2821) from Aebp2 isoform 3/4 are detected in the forward experiment (top) and the reverse experiment (bottom).

F MS/MS spectra indicating the N-terminal peptide of Aebp2 isoform 3/4 : mDIDSTISSGR.

G Western blot of whole-cell lysate of mES cells grown in 2i and serum media. Top: anti-Aebp2; bottom: anti-GAPDH. Arrows A and B show two bands detected by Aebp2 antibody. The intensities of band A and B were compared between 2i and serum normalized to GAPDH as the loading control.

© 2020 The Authors

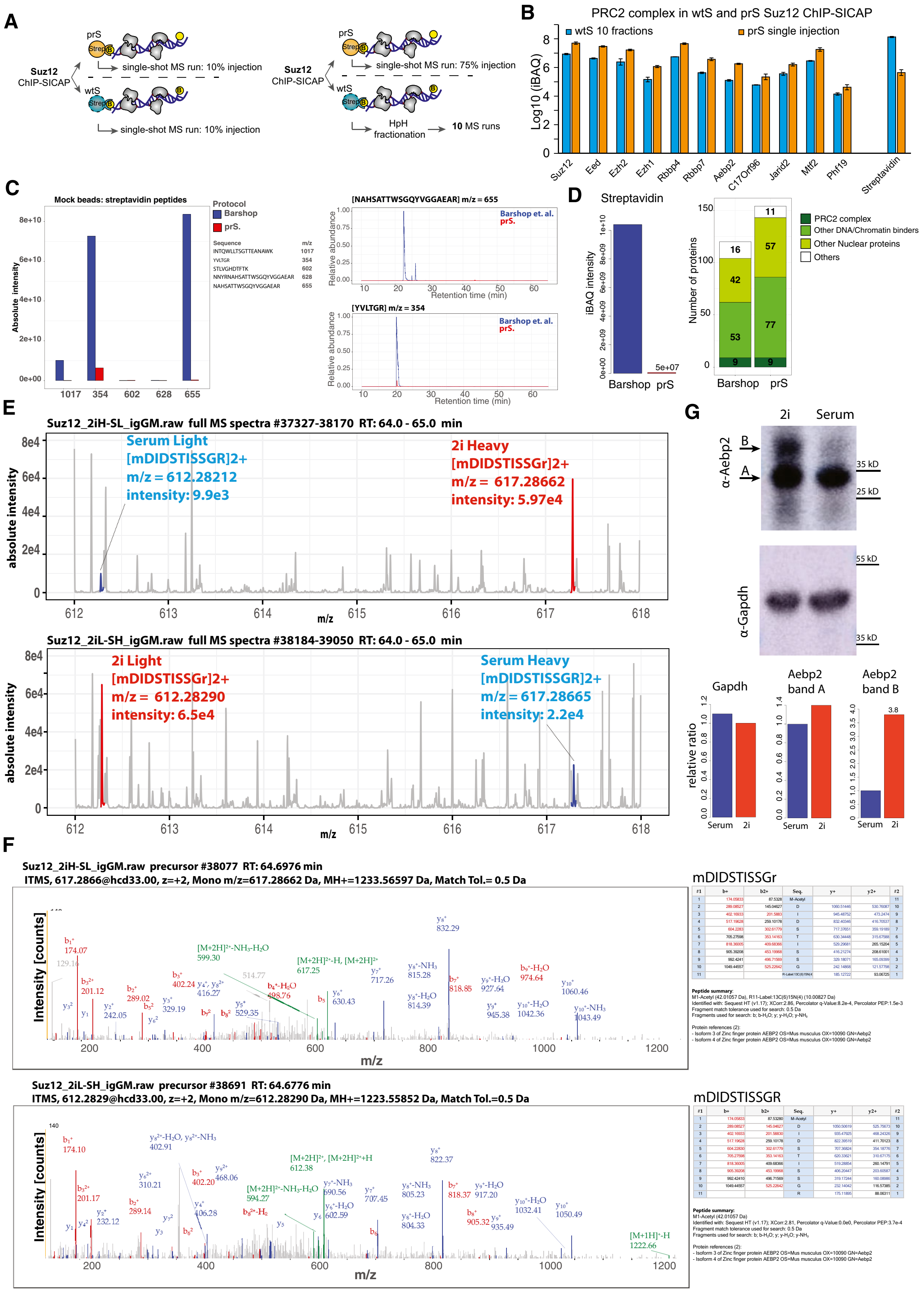
A
Suz12 ChIP-SICAP
prS
single-shot MS run: 10% injection
wtS
single-shot MS run: 10% injection
Suz12 ChIP-SICAP
prS
single-shot MS run: 75% injection
wtS
HpH fractionation
10 MS runs
B
PRC2 complex in wtS and prS Suz12 ChIP-SICAP
wtS 10 fractions
prS single injection
Log10 (iBAQ)
Suz12
Eed
Ezh2
Ezh1
Rbbp4
Rbbp7
Aebp2
C17Orf96
Jarid2
Mtf2
Phf19
Streptavidin
C
Mock beads: streptavidin peptides
Absolute intensity
Protocol
Barshop
prS.
[NAHSATTWSGQYVGGAEAR] m/z = 655
Relative abundance
Retention time (min)
Barshop et. al.
prS.
[YVLTGR] m/z = 354
D
Streptavidin
iBAQ intensity
5e+07
Barshop
prS
Number of proteins
PRC2 complex
Other DNA/Chromatin binders
Other Nuclear proteins
Others
E
Suz12_2iH-SL_igGM.raw full MS spectra #37327-38170 RT: 64.0 - 65.0 min
absolute intensity
Serum Light
[mDIDSTISSGR]2+
m/z = 612.28212
intensity: 9.9e3
2i Heavy
[mDIDSTISSGr]2+
m/z = 617.28662
intensity: 5.97e4
m/z
Suz12_2iL-SH_igGM.raw full MS spectra #38184-39050 RT: 64.0 - 65.0 min
2i Light
[mDIDSTISSGr]2+
m/z = 612.28290
intensity: 6.5e4
Serum Heavy
[mDIDSTISSGR]2+
m/z = 617.28665
intensity: 2.2e4
F
Suz12_2iH-SL_igGM.raw precursor #38077 RT: 64.6976 min
ITMS, 617.2866@hcd33.00, z=+2, Mono m/z=617.28662 Da, MH+=1233.56597 Da, Match Tol.= 0.5 Da
Intensity [counts]
m/z
mDIDSTISSGr
Suz12_2iL-SH_igGM.raw precursor #38691 RT: 64.6776 min
ITMS, 612.2829@hcd33.00, z=+2, Mono m/z=612.28290 Da, MH+=1223.55852 Da, Match Tol.=0.5 Da
mDIDSTISSGR
G
2i
Serum
α-Aebp2
B
A
35 kD
25 kD
55 kD
α-Gapdh
35 kD
Gapdh
Aebp2 band A
Aebp2 band B
relative ratio
3.8
Serum
2i

Figure EV2.

Figure EV3. Comparing performance of prS and wtS beads in biotin-based enrichment experiments. BioID.

A Experimental procedure.

B, C Ranking of proteins identified in BioID experiments performed with either prS or wt beads in target (SEC61B) or control cells and injection of 100 ng (low amount) (B) or 1 μg (high amount) (C).

D The boxplots indicate the number of MS events, the percentage of MS/MS identified, and the peptide-spectrum matches (PSMs) in the different experiments. In the boxplots, the boxes show interquartile ranges (IQR = Q3-Q1). The whiskers extend to data points that are < 1.5 × IQR away from Q1 or Q3 (Turkey whiskers), and the central lines show the medians. *T*-test *P*-values were calculated where **P* < 0.05, ** = 0.001, *** < 0.001.

E, F Integral membrane proteins. (E) Experimental procedure. (F) Intensity and iBAQ values of streptavidin peptides after injecting 10% of the wtS sample, 10% of the prS sample or 90% of the prS sample.

G, H Lipid-interacting proteins. (G) Experimental procedure. (H) Histograms of proteins that were enrichment over control upon biotinylated sphingolipid pull-down with prS beads and on-bead digestion.

 © 2020 The Authors

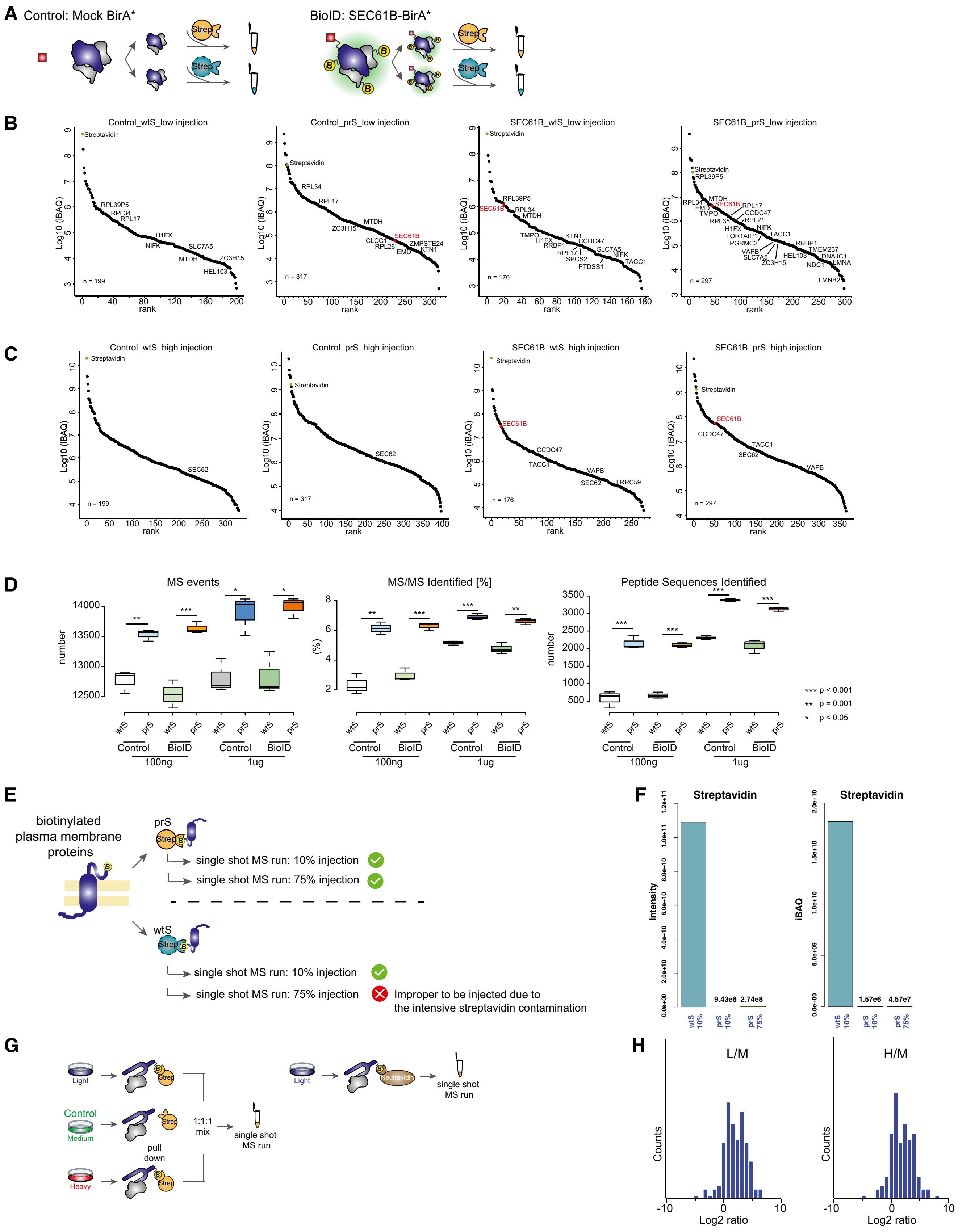
A
Control: Mock BirA*
BioID: SEC61B-BirA*
Strep
B
Control_wtS_low injection
Control_prS_low injection
SEC61B_wtS_low injection
SEC61B_prS_low injection
Log10 (iBAQ)
rank
C
Control_wtS_high injection
Control_prS_high injection
SEC61B_wtS_high injection
SEC61B_prS_high injection
D
MS events
MS/MS Identified [%]
Peptide Sequences Identified
*** p < 0.001
** p = 0.001
* p < 0.05
Control
BioID
100ng
1ug
E
biotinylated plasma membrane proteins
prS
single shot MS run: 10% injection
single shot MS run: 75% injection
wtS
Improper to be injected due to the intensive streptavidin contamination
F
Streptavidin
Intensity
iBAQ
G
Light
Control
Medium
Heavy
pull down
1:1:1 mix
single shot MS run
Neutravidin
H
L/M
H/M
Counts
Log2 ratio

Figure EV3.
